# Weight loss independent outcomes in type 2 diabetes mellitus and other metabolic comorbidities after Roux-en-Y gastric bypass and sleeve gastrectomy

**DOI:** 10.1038/s41366-025-02011-0

**Published:** 2026-03-18

**Authors:** Suzanne Hedberg, Erik Näslund, Johan Ottosson, Erik Stenberg

**Affiliations:** 1 Department of Surgery, Department of Clinical Sciences, The Sahlgrenska Academy, University of Gothenburg, Gothenburg, Sweden; 2 Department of Surgery (Östra Sjukhuset), Sahlgrenska University Hospital, Gothenburg, Sweden; 3 Department of Clinical Sciences, Danderyd Hospital, Karolinska Institutet, Stockholm, Sweden; 4 Department of Surgery, Faculty of Medicine and Health, Örebro University, Örebro, Sweden

**Keywords:** Bariatric surgery, Type 2 diabetes, Obesity, Metabolic syndrome

## Abstract

**Background/objectives:**

Studies show equal or better resolution of type 2 diabetes mellitus (T2D) and other metabolic outcomes after Roux-en-Y gastric bypass (RYGB) compared to sleeve gastrectomy (SG), but it is unclear whether this is related only to the higher weight loss after RYGB, or if there are weight-loss-independent factors. The objective of this study was to examine weight-loss-independent differences in metabolic outcomes between RYGB and SG.

**Methods:**

This study utilized the Scandinavian Obesity Surgery Registry and the Swedish National Diabetes Register. All included patients had presurgical T2D and matching was between RYGB or SG using a 1:1 propensity score, matching with a generalized linear model including age, sex, BMI at baseline, comorbidities (cardiovascular, dyslipidemia, sleep apnea, and hypertension), T2D parameters at baseline (HbA1c, number of T2D medications, insulin use, duration of T2D), year of surgery and percentage Total Weight Loss (%TWL) at nadir. The ensuing cohort was compared regarding remission and improvements in T2D, and other cardiometabolic outcomes, including major adverse cardiovascular events (MACE).

**Results:**

1440 individuals (720 RYGB; 720 SG) were matched 1:1 using Propensity score. There were 494 (68.6%) patients in complete T2D remission at 2 years after RYGB, and 438 (60.8%) after SG, (OR: 0.75, 95% CI 0.60 – 0.93, p = 0.010) despite similar TWL (Standardized mean difference 0.12). SG also had a lower rate of pharmacological remission for T2D (OR 0.71, 95% CI 0.56-0.88, p = 0.002), and hypertension remission (OR 0.70, 95% CI 0.52-0.94, p = 0.019), but there was no significant difference in pharmacological remission regarding dyslipidemia (OR 0.83, 95%CI 0.66-01.04, p = 0.11). No difference was seen in the risk for MACE (SG vs. RYGB HR:1.45, 95%CI 0.89-2.38, p = 0.136).

**Conclusions:**

RYGB is associated with a greater rate of T2D remission compared to SG. This study suggests that these improved outcomes are independent of the degree of weight loss.

## Introduction

Obesity is a disease with rising prevalence worldwide [1–4], that is associated with increased mortality, and with several comorbidities [3]; including type 2 diabetes mellitus (T2D), cardiovascular disease, sleep apnea and musculoskeletal disorders. Metabolic and bariatric surgery (MBS) is an effective, established treatment with improved long-term outcomes regarding both obesity, T2D and other comorbidities [5, 6]. The effects of MBS on T2D are improved glycemic control, often with fewer or no medications, and sometimes complete remission [7].

The two most common MBS procedures are Roux-en-Y gastric bypass (RYGB) and sleeve gastrectomy (SG) [8]. Despite several comparative studies there is no clear scientific view on whether the T2D outcomes differ between RYGB and SG, although, often underpowered studies suggest RYGB may be more effective than SG in the treatment of T2D [7, 9–15]. Although RYGB leads to slightly greater weight loss than sleeve gastrectomy (SG), it remains unclear whether differences in type 2 diabetes (T2D) remission between the procedures are solely attributable to variations in weight loss or if additional weight-independent factors contribute. RYGB involves bypassing the foregut leading to more rapid nutrient delivery to the distal small intestine, which alters the postprandial gastrointestinal hormone response. These changes may contribute to distinct metabolic effects beyond weight reduction [16–18]. In contrast, SG also accelerates nutrient flow over the pylorus, but the resulting changes in postprandial gastrointestinal hormone response differ, with some (e.g., GLP-1) being less pronounced than those observed in RYGB [17–19].

The aim of this registry-based retrospective study was to compare the effects of RYGB and SG, independent of weight loss, on the outcome of T2D, and of cardiometabolic outcomes, in a closely matched cohort of patients who underwent RYGB or SG.

## Methods

This study is based on a matched cohort of patients with severe obesity and T2D included both in the Swedish National Diabetes Register (NDR) and the Scandinavian Obesity Surgery Registry (SOReg). NDR is a national quality register that started in 1996 and covers diagnostic information and clinical data on nearly all patients with diabetes in Sweden, with data obtained from outpatients settings in both primary care and specialized clinics [20]. SOReg, a national quality and research registry, started in 2007 and covers virtually all MBS in Sweden, with a high validity of data [21]. Using the personal identification numbers, patients in NDR and SOReg can be linked, in these registries as well as with the Total Population Registry, that covers all mortality and migration [22].

### Inclusion

Patients considered for inclusion were adults (>18 years of age) with T2D prior to undergoing a primary RYGB or SG in 2007–2021, for whom HbA1c at baseline and at weight loss nadir (the highest weight-loss as measured at 1- or 2-year follow-up visits postoperatively) was registered. Patients who had had revisional surgery, or had died or emigrated before 2 years of follow-up were excluded.

### Matching

Patients with T2D who underwent a RYGB were matched with patients who underwent a SG using a 1:1 propensity score matching with a generalized linear model including age, sex, BMI at baseline, comorbidities (cardiovascular, dyslipidemia, sleep apnea, and hypertension), T2D parameters at baseline (HbA1c, number of T2D medications, insulin use, duration of T2D), year of surgery and Total Weight Loss (TWL) at nadir.

### Definitions

T2D at baseline was defined as diagnosis and the use of one or more pharmacological treatments. Complete remission was defined as a HbA1c of 48 mmol/mol (6.5%) or less without pharmacological treatment [23]. Pharmacological remission of T2D, hypertension or dyslipidemia was defined as the cessation of pharmacological treatment for each condition (Supplementary Table S1). Previous alcohol use disorder was defined as a contact with specialized care or the use of pharmacological treatment for this condition. Active alcohol use disorder was considered a contraindication for surgery. TWL at nadir was defined as the lowest recorded weight loss, at 1 or 2 years follow up ((weight at nadir—weight before preoperative weight reduction)/weight before preoperative weight reduction); while this may not be the exact nadir for each patient it captures the TWL in the time period of nadir, and in the same way in both groups.

### Outcomes

The main outcome measure was complete remission of T2D. Secondary outcome measures were pharmacological remission, change in number of T2D medications, differences in HbA1c, and the cessation of insulin use, as well as differences in improvements in dyslipidemia, hypertension, mortality, risk for major adverse cardiac events (MACE), fractures, and alcohol use disorders. MACE was defined as the first occurrence of an acute coronary event, cerebrovascular event or all-cause mortality (Supplementary Table S2).

### Statistics

Continuous variables are presented as means with standard deviations or medians with Interquartile range where appropriate, whereas categorical values are presented numerically with proportions. The balancing of the propensity score matching was evaluated using a standardized mean difference, with a difference of more than 0.1 signifying a residual imbalance.

The outcomes were evaluated using descriptive statistics, and logistic and Cox regression models as appropriate. The values that remained imbalanced after propensity score matching were included in the models, to account for the residual imbalance. The secondary analysis for alcohol use disorders was also adjusted for previous alcohol use.

A p-value of less than 0.05 was considered statistically significant.

IBM SPSS Statistics version 29, and R Studio version 4.2.0 (R Foundation for Statistical Computing) were used for statistical analysis.

### Ethics

This study was approved by the Swedish ethical review authority (ref.no. 2022-05359-01) and was conducted in accordance with the Helsinki Declaration and its amendments [24]. In accordance with current Swedish legislation, and the Swedish ethical review authority patients may opt-out of SOReg och NDR at any time, but informed consent is not required for the current study.

## Results

The initial group considered for inclusion consisted of 6007 patients, 5249 RYGB and 758 SG. After propensity score matching the group consisted of 1440 patients: 720 RYGB and 720 SG. The baseline characteristics as well as TWL at nadir of the included patients are shown in Table 1. All baseline characteristics were well balanced, with a small residual imbalance in TWL nadir only. Mean postoperative follow-up was 5.2 (2.5) years.

**Table 1:** Baseline characteristics after propensity score matching.

	*Roux-en-Y Gastric Bypass*	*Sleeve Gastrectomy*	*Standardized mean difference*
*Number*	720	720	-
*Age(years)*	48.6 ± 10.08	48.9 ± 9.96	0.029
*Body Mass Index (kg/m*^2^*)*	41.0 ± 5.74	40.8 ± 5.23	0.040
*Women*	411 (57.1%)	429 (59.6%)	0.051
*Obstructive Sleep Apnea*	128 (17.8%)	126 (17.5%)	0.007
*Hypertension*	444 (61.7%)	434 (60.3%)	0.028
*Cardiovascular comorbidity*^a^	77 (10.7%)	85 (11.8%)	0.035
*Chronic Obstructive Pulmonary Disease*	28 (3.9%)	22 (3.1%)	0.045
*Dyslipidemia*	340 (47.2%)	329 (45.7%)	0.030
*Previous Alcohol use disorder*	23 (3.2%)	28 (3.9%)	0.038
*Insulin treatment*	182 (25.3%)	158 (21.9%)	0.078
*Number of diabetes drugs*	1.5 ± 0.75	1.5 ± 0.75	0.050
*HbA1c*	56.2 ± 14.64	55.7 ± 14.69	0.037
*Duration of Diabetes (years)*	5.2 ± 5.04	5.3 ± 5.46	0.018
*Total weight loss at nadir(%)*	25.6 ± 7.25	24.6 ± 8.29	0.120

Presented as numbers (proportion) or mean ± standard deviation.

^a^Previous ischemic heart disease, heart failure or arrhythmic heart disease.

### Type 2 diabetes mellitus

There were 494 (68.6%) patients in complete T2D remission at 2 years after RYGB, and 438 (60.8%) after SG, corresponding to a lower rate of complete remission after SG compared to RYGB (adjusted-OR: 0.75, 95%CI 0.60 – 0.93, p = 0.010) after the same weight loss. There were 512 (71.1%) patients in pharmacological remission (cessation of use of glucose-lowering medications) after RYGB and 449 (62.4%) after SG (adjusted-OR 0.71, 95%CI 0.56-0.88, p = 0.002). The HbA1c at nadir was 40.8 (9.3) for RYGB and 41.75 (11.2) for SG, p = 0.07.

**Fig. 1:**
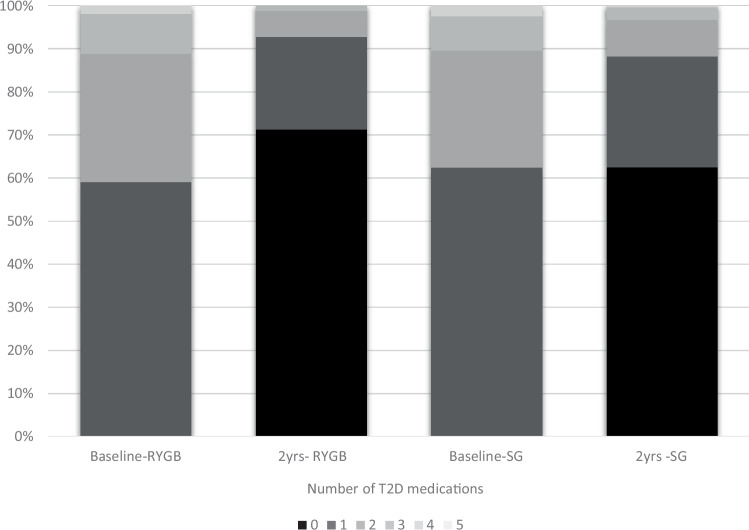
Proportions of number of medications for type 2 Diabetes (T2D) at baseline and 2 years for patients receiving Roux-en-Y Gastric Bypass or Sleeve Gastrectomy. The colors show the number of medications at the different timepoints for RYGB and SG (darker color-fewer medications), visualizing both the improvement seen after both surgeries, and the difference between the groups.

The mean number of T2D medications at 2-year follow-up was 0.37 (0.66) for RYGB and 0.53 (0.80) for SG, p < 0.001. The proportional change in the number of mediations used at baseline and at 2 years for RYGB and SG are shown in Fig. 1. While a similar tendency was seen irrespective of numbers of glucose-lowering medications at baseline, a higher reduction in mean number of glucose-lowering medications was seen after RYGB compared to SG among patients having at least 2 glucose-lowering medications at baseline (Table 2).

**Table 2:** The change in mean number of T2D medications and Odds Ratio of pharmacological remission for SG compared to RYGB depending on number of T2D medications at baseline.

	*RYGB*		*SG*			*Odds ratio, adjusted for TWLnadir*		
No. of medications, base-line	No. of patients	Mean no. of medications	No. of patients	Mean no. of medications	p-value, means	OR	95% CI	p-value
1	423 (48.5%)	0.23 (0.48)	449 (51.5%)	0.30 (0.52)	0.057	0.73	0.53-1.00	0.05
2	216 (52.6%)	0.50 (0.77)	195 (47,4%)	0.73 (0.94)	0.007	0.71	0.48-1.07	0.10
≥3	81 (51.6%)	0.77 (0.86)	76 (48.4%)	1.41 (1.04)	<0.001	0.28	0.13-0.58	<0.001

Mean (SD).

Of the 340 patients that used insulin at baseline (182 (53.5%) RYGB and 158 (46.5%) SG), 244, had been able to stop insulin usage at 2 years (139 (76.4%) RYGB and 105 (66.5%) SG. The chance of insulin cessation was higher, although not statistically significant in RYGB compared to SG (adjusted-OR 1.59, 95%CI (0.99–2.56), p = 0.057).

### Hypertension

There were 878 (61.0%) patients, 444 (61.7%) RYGB and 434 (60.3%) SG, p = 0.59 that used pharmacological treatment for hypertension at baseline. At 2 years, 290 (33.0%) of those patients were in pharmacological remission, 182 (41.0%) RYGB and 108 (24.9%) SG, p < 0.001. The chance of pharmacological remission from hypertension was lower after SG compared to RYGB (adjusted-OR 0.70, 95%CI 0.52–0.94, p = 0.019).

The mean number of medications for treating hypertension at baseline was 1.37 (1.36) for RYGB and 1.38 (1.36) for SG, p = 0.97. At 2 years, the mean numbers had decreased to 0.72 (1.12) for RYGB and 0.89 (1.18) for SG, p = 0.006.

### Dyslipidemia

There were 669 (46.5%) patients, 340 (47.2%) RYGB and 329 (45.7%) SG, p = 0.56, that used pharmacological treatment for dyslipidemia at baseline. At 2 years, 253 (37.8%) of those patients were in pharmacological remission, 149 (43.8%) RYGB and 104 (31.6%) SG, p = 0.048. The chance of pharmacological remission from dyslipidemia was not significantly lower after SG compared to RYGB (adjusted-OR 0.83, 95%CI 0.66–01.04, p = 0.11).

Analysis of triglycerides at 2 years showed lower mean values in RYGB (n = 225, 1.2 (0.53) mmol/l; 132.9 (46.9) mg/dl) compared to SG (n = 263, 1.6 (0.85) mmol/l; 141.7 (75.3) mg/dl, p = <0.001). The corresponding values for LDL are RYGB (n = 267, 2.41 (0.85) mol/l; 93.2 (32.9) mg/dl) and SG (n = 308, 2.95 (1.04) mmol/l; 114.1 (40.2) mg/dl, p < 0.001), and for HDL RYGB (n = 247, 1.40 (0.39) mmol/l; 54.1 (15.1) mg/dl), and SG (n = 285, 1.37 (0.36) mmol/l; 53.0 (13.9) mg/dl, p = 0.41).

### Mortality and major adverse cardiovascular events

During the mean follow-up time (5.2 (2.5) years), 14 (incidence rate 3.84, 95%CI 2.10–6.44/1000 person-years) RYGB patients died and 28 (incidence rate 7.74, 95%CI 5.14–11.19/1000 person-years) suffered from at least one episode of MACE while 18 SG patients (incidence rate 4.77, 95%CI 2.83–7.53/1000 person-years) died and 39 (incidence rate 10.53, 95%CI 7.49-14.39/1000 person-years) suffered from at least one episode of MACE (adjusted-hazard ratio for SG vs RYGB for mortality 1.36, 95%CI 0.67–2.77, p = 0.395; adjusted-hazard ratio for MACE 1.45, 95%CI 0.89–2.38, p = 0.136). A non-fatal acute cardiovascular event was seen in 9 (incidence rate 2.48, 95%CI 1.13–4.71/1000 person-years) RYGB patients and 13 (incidence rate 3.49, 95%CI 1.86–5.97/1000 person-years) SG patients (adjusted-hazard ratio 0.71, 95%CI 0.30–1.66, p = 0.428), while a non-fatal cerebrovascular event was seen in 5 (incidence rate 1.37, 95%CI 0.45–3.21/1000 person-years)RYGB patients and 12 (incidence rate 3.20, 95%CI 1.65–5.59/1000 person-years) SG patients (adjusted-hazard ratio 2.41, 95%CI 0.84–6.87, p = 0.100).

### Fractures and alcohol use disorders

During follow-up, 34 SG patients (incidence rate 9.26, 95%CI 6.41–12.94/1000 person-years) patients suffered from at least one fracture compared to 51 RYGB patients (incidence rate 14.65, 95%CI 10.91–19.26/1000 person-years; adjusted hazard ratio 0.62, 95%CI 0.40–0.97; p = 0.035). Treatment for alcohol use disorder occurred in 18 patients after SG (incidence rate 4.86, 95%CI 2.88-7.68/1000 person-years) and 34 patients after RYGB (incidence rate 9.63, 95%CI 6.67–13.47/1000 person-years; adjusted HR 0.45, 95%CI 0.25–0.79; p < 0.006).

## Discussion

Patients with obesity and T2D operated with RYGB showed better outcomes in terms of both complete and pharmacological remission of T2D, as well as pharmacological remission of dyslipidemia and hypertension, but more often had fractures and alcohol use disorder compared to those who underwent SG. This study suggests that these differences in outcomes are in part independent of the degree of weight loss.

Although both RYGB and SG are effective treatments for obesity, they operate through somewhat different mechanisms [25]. Previous studies have reported differing postprandial responses in gastrointestinal hormones such as ghrelin, cholecystokinin, glucagon-like-peptide 1 (GLP-1) and gastrointestinal peptide (GIP). Sleeve gastrectomy is associated with reduced fasting and postprandial ghrelin and cholecystokinin responses, while RYGB leads to a more pronounced GLP-1 response to meals and reduced GIP response [17–19]. These variations in gastrointestinal hormonal responses may contribute to the weight loss independent effects observed in this study. However, differences in outcome related to the surgical procedure itself are difficult to investigate, as several other factors independently influence the likelihood of T2D remission, including the known differences in weight loss, which have previously been speculated to confound the results. The matched design used in this study provides a unique opportunity to examine procedure-related outcomes while controlling for other influential factors, such as total weight loss.

Earlier studies have suggested that RYGB may have additional weight-loss-independent effects, but many lacked sufficient statistical power to detect differences between procedures [7] or did not fully account for the differences in weight loss [26], even though analyses indicated both weight-dependent and weight-independent components. Interestingly, Jans et al. also found a higher risk of relapse in T2D following SG-induced remission [27], which supports the notion of a weight-loss independent mechanism for improvements in and resolution of cardiometabolic disorders after RYGB compared to SG.

Previous comparisons of mid- to long-term outcomes after RYGB and SG have generally reported better metabolic results following RYGB, though it is also associated with greater weight loss [9, 28]. Interestingly, in an investigation similar to ours, a retrospective matched analysis of T2D outcomes, albeit with a lower number of patients at fewer surgical facilities, Hage et al. also report better T2D results following RYGB than SG [29]. While our study showed higher remission rates than those reported by Hage et al. for both SG (60.8% vs 33%) and RYGB (68.6% vs 46.4%), the Hage patients were older, with a higher BMI at time of surgery which may explain the lower rates of remission. There was, however, also a longer follow-up, in the study by Hage et al. and the lower remission rates may reflect an increased relapse in T2D over time, seemingly more so in the SG than in the RYGB group. The finding of the current study suggest that there are additional weight-independent mechanisms favoring RYGB. Furthermore, these results indicate that patients with more advanced metabolic disease may benefit the most from RYGB. This consideration is particularly important when selecting a surgical approach for patients with advanced metabolic disease, especially given the favorable safety profiles of both procedures [30].

Although the improved metabolic outcomes with RYGB did not translate into better MACE or mortality in the present study, possibly due to limited number of patients and a relatively short follow-up, these outcomes should be a focus of future research. Preventing micro- and macrovascular complications remain a central goal in treating obesity in patients with T2D who undergo MBS.

The medium to long-term nutritional and psychiatric side effects of these metabolic benefits have been previously recognized [31–33]. The risk for fractures is likely associated with variations in nutrient absorption combined with increased bone turnover [31], factors that are probably mostly independent of weight loss. Similarly, differences in alcohol absorption and the risk of alcohol use disorder are likely to be primarily driven by weight-independent mechanisms. These negative side effects should be carefully considered in light of the metabolic effects of RYGB, particularly for patients at increased risk of fractures or alcohol use disorder, even among those with T2D.

The positive weight-independent factors must be considered in light of the negative malabsorptive and psychological side effects of RYGB compared to SG. This knowledge should be considered in the evaluation of the individual patient for whom the choice of procedure should be adapted to the condition, metabolic profile, comorbidities risks, and expectations of the individual patient.

Strengths of this study include the high validity and quality of data from the registries as well as the closely matched design. While it is a retrospective observational study and cannot define causation it lends further strength to the prior studies [9, 34–36], showing that RYGB has a more substantial effect on metabolic comorbidities than SG and suggests that this effect is, at least in part, weight loss independent. While Swedish law prohibits the registration of race, the Swedish population is predominantly white and thus the metabolic outcomes of other ethnic populations may differ. The study is also limited to the patients who fulfilled at least 2 years of follow-up; however, the combination of the registries does mean that the proportion of patients lost is small, and similar in both RYGB and SG. The use of pharmacological remission is also a limiting factor as several medications can be used with multiple indications, i.e., metformin for polycystic ovary syndrome, as well as T2D, or metoprolol for cardiac protection and hypertension. Pharmacologic remission may also be problematic as some preventive measures may be hard to discontinue once started, which may be the case for some dyslipidemia medication. However, these limitations do rather suggest that the outcomes may in fact be even better than shown, and there are no data to suggest a difference between groups. Other unknown factors may also contribute to residual confounding, which is challenging to eliminate in registry-based epidemiological research.

In conclusion, this study indicates that patients achieve better metabolic outcomes after RYGB compared to SG and suggests that these outcomes are, at least in part, independent of weight loss.

## Supplementary information

Supplementary tables

## Data Availability

Data cannot be shared publicly due to patient confidentiality under Swedish legislation. Data are available from the Scandinavian Obesity Surgery Registry (soreg@regionorebrolan.se), the National Diabetes Register (ndrinfo@registercentrum.se) and the Swedish Board of Health and Welfare (Registerservice@socialstyrelsen.se).
