# Supplementary material for: Weight loss independent outcomes in type 2 diabetes mellitus and other metabolic comorbidities after Roux-en-Y gastric bypass and sleeve gastrectomy
Source: Int J Obes (Lond). 2026 Mar 18;50(5):1158–63. doi: 10.1038/s41366-025-02011-0 (PMC13226073; doi:10.1038/s41366-025-02011-0)
Supplement: Supplementary file 1 — Supplementary tables [file 41366_2025_2011_MOESM1_ESM.pdf]

**Table S1**. Definition of baseline variables		
Variable	Definition	Source
Age	Time from birth until surgery	SOReg
Sex	Biological sex	SOReg
Body Mass Index	Value before preoperative weight reduction	SOReg
Obstructive sleep apnea	CPAP/BiPAP treatment	SOReg
Hypertension	Pharmacological treatment for hypertension	SOReg, National Prescribed drug register
Cardiovascular comorbidity	Previous diagnosis of ischemic heart disease (ICD-10: I20-22), heart failure (ICD-10: I50) or arrhythmic heart disease (ICD-10: I47-48)	National patient registry
Chronic obstructive pulmonary disorder	Admission for COPD or a complication of COPD with COPD as secondary diagnosis, or prescription of an anticholinergic drug (ATC: R03BB), long-acting beta-2 agonist (ATC: R03AC12-R03AC18) or a combination of these (ATC: R03AL)	National patient registry, National Prescribed drug register
Dyslipidemia	Pharmacological treatment for dyslipidemia	SOReg, National Prescribed drug register
Insulin treatment	Insulin treatment (ATC: A10) within 12 months before surgery	National Prescribed drug register
Number of drugs	Number of different treatments for diabetes combined at the time of surgery	National Prescribed drug register
HbA1c	HbA1c value before preoperative weight reduction	NDR, SOReg
Duration of type-2 diabetes	Years since diagnosis of type-2 diabetes	NDR, SOReg, National patient registry, National Prescribed drug register
Weight loss	Difference in weight at follow-up – weight before preoperative weight reduction	NDR, SOReg
Previous alcohol use disorder	Specialized care (ICD-10: F10)<br>Dispensed drugs (ATC: N07BB)	National patient registry; National Prescribed drug register

**Table S2**. Definitions of outcome variables		
Variable	Definition	Source
Major adverse cardiovascular event (MACE)	Specialized care (ICD-10: I20.0, I21-22, I60, I61, I63) or all-cause mortality	National patient registry
Acute coronary syndrome	Specialized care (ICD-10: I20.0, I21-22)	National patient registry
Cerebrovascular event	Specialized care (ICD-10: I60,61,63)	National patient registry
All-cause mortality	Death of any cause	Total population registry
Remission of type-2 diabetes	normalized blood samples (HbA1c <48 mmol/mol) without pharmacological treatment (ATC: A10) during a 12-month period	NDR, SOReg, National Prescribed drug register
Insulin use	Dispensed insulin (ATC: A10A) during a 12-month period	National Prescribed drug register
Pharmacologic treatment for dyslipidemia	Dispensed drugs (ATC: C10) during a 12-month period	National Prescribed drug register
Pharmacologic treatment for hypertension	Dispensed drugs (ATC: C02, C03, C07, C08, C09) during a 12-month period	National Prescribed drug register
Fractures	Specialized care (ICD-10: S32, S42, S52, S62, S72)	National patient registry
Alcohol use disorders	Specialized care (ICD-10: F10) Dispensed drugs (ATC: N07BB)	National patient registry; National Prescribed drug register
